# Choosing algorithms for TB screening: a modelling study to compare yield, predictive value and diagnostic burden

**DOI:** 10.1186/1471-2334-14-532

**Published:** 2014-10-19

**Authors:** Anna H van’t Hoog, Ikushi Onozaki, Knut Lonnroth

**Affiliations:** Academic Medical Centre, Department of Global Health, University of Amsterdam, Amsterdam, The Netherlands; Amsterdam Institute for Global Health and Development, Amsterdam, The Netherlands; Global TB Programme, World Health Organization, Geneva, Switzerland

**Keywords:** Tuberculosis, Systematic screening, Diagnostic algorithms, Modelling

## Abstract

**Background:**

To inform the choice of an appropriate screening and diagnostic algorithm for tuberculosis (TB) screening initiatives in different epidemiological settings, we compare algorithms composed of currently available methods.

**Methods:**

Of twelve algorithms composed of screening for symptoms (prolonged cough or any TB symptom) and/or chest radiography abnormalities, and either sputum-smear microscopy (SSM) or Xpert MTB/RIF (XP) as confirmatory test we model algorithm outcomes and summarize the yield, number needed to screen (NNS) and positive predictive value (PPV) for different levels of TB prevalence.

**Results:**

Screening for prolonged cough has low yield, 22% if confirmatory testing is by SSM and 32% if XP, and a high NNS, exceeding 1000 if TB prevalence is ≤0.5%. Due to low specificity the PPV of screening for any TB symptom followed by SSM is less than 50%, even if TB prevalence is 2%. CXR screening for TB abnormalities followed by XP has the highest case detection (87%) and lowest NNS, but is resource intensive. CXR as a second screen for symptom screen positives improves efficiency.

**Conclusions:**

The ideal algorithm does not exist. The choice will be setting specific, for which this study provides guidance. Generally an algorithm composed of CXR screening followed by confirmatory testing with XP can achieve the lowest NNS and highest PPV, and is the least amenable to setting-specific variation. However resource requirements for tests and equipment may be prohibitive in some settings and a reason to opt for symptom screening and SSM. To better inform disease control programs we need empirical data to confirm the modeled yield, cost-effectiveness studies, transmission models and a better screening test.

**Electronic supplementary material:**

The online version of this article (doi:10.1186/1471-2334-14-532) contains supplementary material, which is available to authorized users.

## Background

The current global rate of decline in TB incidence, at about 2% annually, is grossly insufficient to reach the goal of TB elimination by 2050 [1]. Missed and delayed diagnosis of active TB helps sustain transmission and is a major contributor to the slow rate of decline. It also translates into poor health outcomes in people who get no or too late access to appropriate treatment [2]. In order to improve early detection of TB, active case finding approaches may be needed in certain risk groups with high TB prevalence or poor access to TB diagnosis. A recently released WHO guideline provides recommendations on when, whom and how to screen for active TB [3, 4].

TB screening is defined as “systematic identification of people with suspected active TB in a predetermined target group, using tests, examinations, or other procedures which can be applied rapidly” [3]. Screening is offered systematically to predetermined target groups, and not only to individuals seeking care for symptoms or signs [4]. Screening could target both people who seek health care (with or without symptoms/signs consistent with TB) and people who do not seek care. In addition to prerequisites that need to be met regarding TB program performance and health system capacity, the decisions about screening require prioritization of risk groups, an approach on how to reach the intended populations, and choices with respect to the screening and diagnostic algorithm [3].

**Figure 1:**
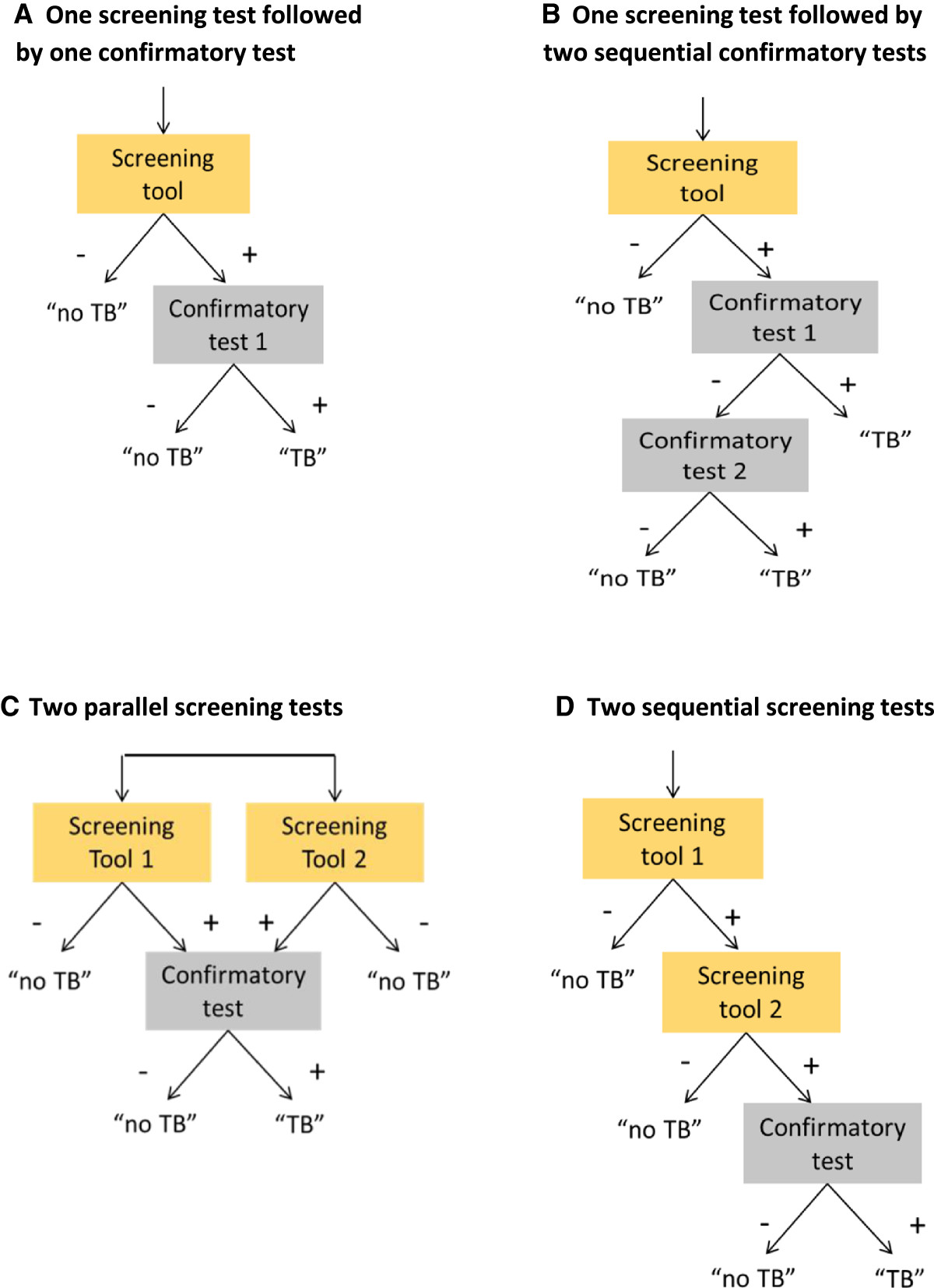
**Algorithms composed of one or more screening methods and one or more confirmatory tests.** In panel **A** one screening tool is applied (e.g. symptoms) and screen positives are further evaluated by one confirmatory test with high sensitivity and high specificity (e.g. Xpert MTB/RIF). In panel **B** one screening tool is applied (e.g. symptoms) and screen positives are further evaluated by a confirmatory test with low sensitivity (e.g. sputum smear microscopy), and persons with a negative test receive a second test or procedure (e.g. clinical diagnosis, or sputum culture). In panel **C** two screening tools are applied (e.g. symptoms and chest radiography) and screen positives on either one or on both are further evaluated with a confirmatory test. In panel **D** two screening tools are applied sequentially. Screen positives on the first screen (e.g. symptoms) undergo a second screen (e.g. CXR) and if also positive on the second a confirmatory test is applied. The single confirmatory test in panels **C** and **D** could also be replaced by two-steps as in panel **B**.

Such an algorithm will generally be composed of one or more screening methods and one or more confirmatory tests (Figure 1). Symptom questionnaires and chest radiography are the most available and best documented methods to screen for active TB disease [5, 6]. Confirmatory tests are historically sputum smear microscopy, and mycobacterial culture, which is the reference standard of diagnostic testing for TB [7], and the more recently developed molecular Xpert MTB/RIF assay (Cepheid, Sunnyvale, CA) [8]. However, culture is often not available for routine diagnosis in high TB burden countries [9], and requires a much longer wait for results (2–6 weeks) than the Xpert MTB/RIF assay (XP) and sputum-smear microscopy (SSM), both of which can provide final test results in less than 1 day [8]. In theory, the algorithm could comprise of one simple and cheap rapid test that can diagnose TB with high sensitivity and very high specificity. However, in practice none of the currently available tests for TB fulfill this ideal profile [9]. A screening initiative will thus need to consider which algorithm is the most appropriate for a specific risk group in a specific setting. This requires balancing the yield of true- and false-positive and -negative TB, the benefits and risk of each outcome, the resource requirements, cost-effectiveness, and the feasibility of reaching and enabling access to full diagnostic work-up as well as required care for the screened population.

To inform the choice of an appropriate algorithm we discuss clinical epidemiological principles that are important for screening and diagnostic algorithms, and we use a simple mathematical model to compare algorithms that may likely be considered for TB screening using currently available screening and diagnostic methods. We compare the yield, positive predictive value, and requirements in terms of diagnostic tests.

## Methods

### Algorithm

In our calculations we consider a population of 100,000 persons who will be screened. The TB prevalence used in the calculations determines the number of persons in the total population who truly have active pulmonary TB that would be detectable by a highly sensitive and specific bacteriological test like liquid mycobacterial culture. We compare 12 algorithms, composed of one or more screening methods and one or more confirmatory tests [Figure 1]. The 12 combinations of screening methods and confirmatory tests are listed in Table 1.

**Table 1:** **The 12 combinations of screening methods and confirmatory tests**

Number	Screening method		Confirmatory test	
	First	Second (if 1st positive)	First	Second (if 1st negative)
1	Prolonged cough*		SSM	CD^§^
2	Prolonged cough*		XP	CD^§^
3	Prolonged cough*	CXR^‡^	SSM	CD**
4	Prolonged cough*	CXR^‡^	XP	CD**
5	Any TB Symptom+		SSM	CD^§^
6	Any TB Symptom+		XP	CD^§^
7	Any TB Symptom+		SSM	CD^§^
8	Any TB Symptom+	CXR^‡^	XP	CD^§^
9	CXR abnormality suggestive of TB	CXR^‡^	SSM	CD^§^
10	CXR abnormality suggestive of TB		XP	CD^§^
11	Any CXR abnormality		SSM	CD^§^
12	Any CXR abnormality		XP	CD^§^

SSM = Sputum smear microscopy; XP = Xpert MTB/RIF; TB = tuberculosis; CXR = chest X-ray.

CD = clinical diagnosis, which may in addition to clinical judgment include antibiotic trial and/or CXR for TB abnormalities.

*Cough for 2-3 weeks or longer †Any one out of 4-7 symptoms suggestive of TB.

^§^We assume that the proportion of persons who receive a clinical diagnosis depends on the negative predictive value of the prior algorithm, as explained in the Methods.

^‡^Any CXR abnormality.

**All persons with a negative first confirmatory test receive a clinical diagnosis.

Persons with a negative screen would not be further evaluated. Individuals with a positive screening result will require one or more diagnostic tests to establish a final TB diagnosis. Individuals with a positive screen, but negative confirmatory test would either receive a definite diagnosis of no TB, or may be advised on follow up care if warranted.

**Figure 2:**
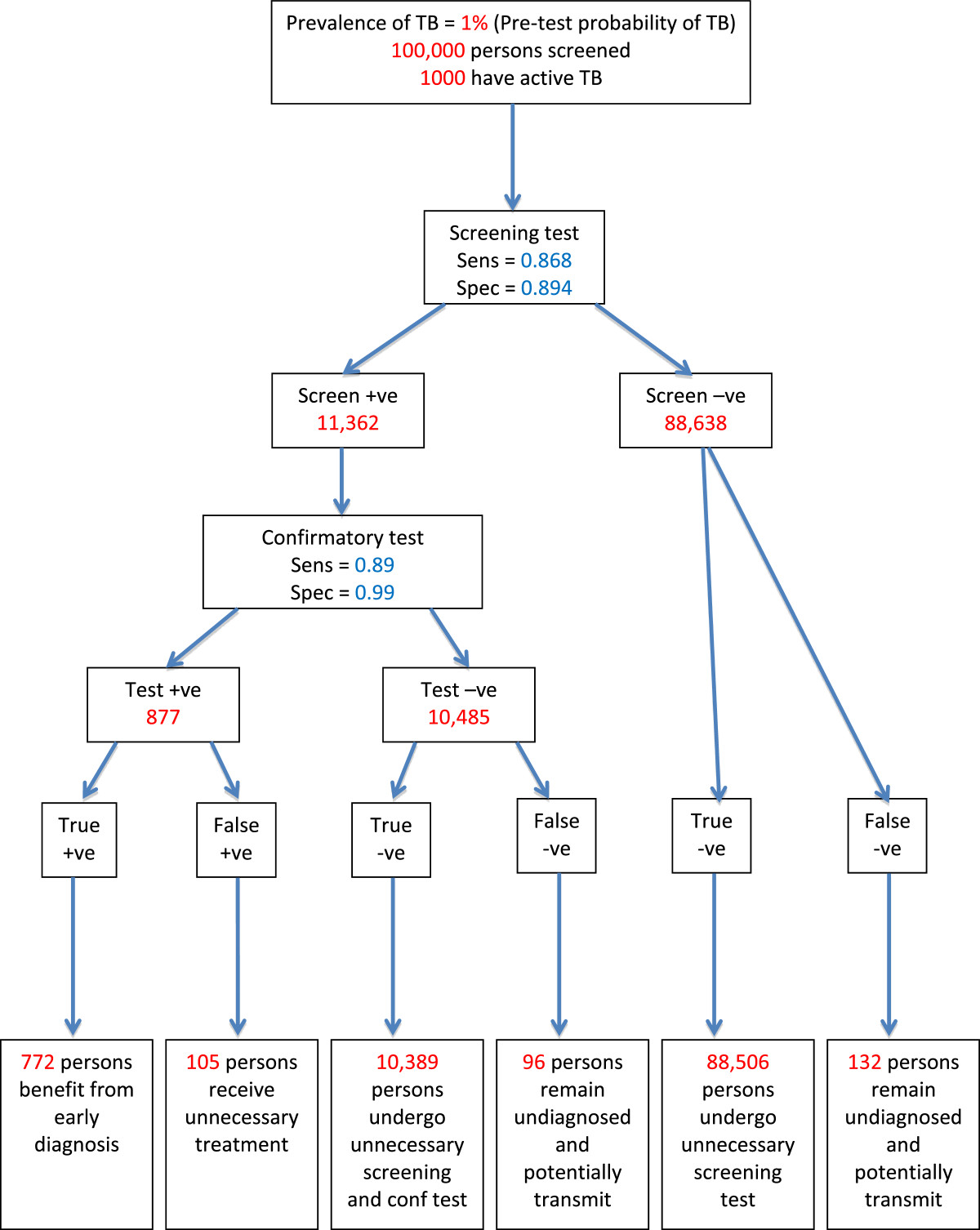
**Outcomes of an example screening and diagnostic algorithm.** Modified after Lonnroth IJTLD 2013. TB = tuberculosis, +ve = positive, -ve = negative.

Six categories of algorithm outcomes are possible (Figure 2):

A. True positives (TP), who are screen-positive, confirmatory test-positive, and truly have active TB. These individuals will benefit from screening provided that they receive proper TB treatment, which would not otherwise have been received or which would have been received after a delay of clinical importance. There is also potential community benefit of this outcome, through reduced TB transmission [10]. The TP may face costs and inconvenience of screening and diagnostic procedures as well as adverse health, financial and social effects of appropriate TB treatment [11, 12].
B. False positives (FP), who are screen-positive, confirmatory test-positive, but do not have active TB. These individuals may face costs and inconvenience of screening and diagnosis as well as adverse health, financial and social effects of unnecessary TB treatment [11, 12].
C. True negatives (TN), who are screen-positive, but confirmatory test-negative, and do not have active TB. These individuals will benefit from knowing that they do not have TB. However, they may face costs and inconvenience of screening and diagnostic procedures that could be regarded as unnecessary. Depending on the type of test, they may be identified as at higher risk to develop TB disease in the future, which may be a benefit or a disadvantage.
D. True negatives, who are screen-negative, and do not have active TB. These individuals will benefit from knowing that they do not have TB. However, they may face costs and inconvenience of screening that could be regarded as unnecessary.
E. False negatives (FN), who are screen-positive, confirmatory test-negative but actually have active TB. These individuals remain undiagnosed due to the limitation of the confirmatory test(s). They may face costs and inconvenience of screening and of the confirmatory test(s).
F. False negatives, who are screen-negative but have active TB. They remain undiagnosed due to the limitation of the screening test(s).

In both E and F, the negative consequences of a FN diagnosis includes false reassurance discouraging care-seeking and delaying later diagnosis. Negative consequences may affect the individual, and others who may become infected.

The extent to which screening correctly identifies all persons with bacteriologically active TB among the population to be screened, i.e. the proportion of cases detected, is determined by the sensitivity of the screening method and of the confirmatory test(s), and by acceptance to participate and adherence to the algorithm. However, we focus on differences that can be expected from different algorithms and assume 100% participation for this study.

The prevalence of active TB in the population to be screened is an important determinant of the tradeoff between benefits, risks and costs. The latter includes financial, logistical and human resource requirements as well as participants’ time and personal expenses. This tradeoff can be judged by the following three indicators, which all depend on the TB prevalence: (i) the number needed to screen (NNS) and thus the resources required to detect one true TB case. Furthermore (ii) the positive predictive value (PPV), i.e. the number of true positive cases detected divided by the sum of true positive cases detected and false-positive TB diagnoses made, and (iii) the negative predictive values (NPV), i.e. the number of true negatives divided by the sum of true negative and false negative outcomes.

### Screening methods

We consider the most available screening methods, which are symptom questions, chest radiography (CXR), and serial screening with first a symptom questionnaire followed by CXR if the symptom screen is positive (Figure 1D). We left out algorithms composed of symptom and CXR screening in parallel (Figure 1C). Although this combination is the standard screening in TB prevalence surveys, it has little advantage for an active screening program as the sensitivity of symptom and CXR screening in parallel is only marginally better compared to the sensitivity of CXR screening for any abnormality alone, and its specificity is worse [5]. The sensitivity and specificity of the symptom screens as used in the model (Table 2) are obtained from a systematic review and meta-analysis [5]. We examine symptom screening for ‘prolonged cough’ i.e. cough for 2-3 weeks or more, and for ‘any TB symptom’ i.e. presence of any one symptom out of a combination of 4-7 symptoms suggestive of TB: cough, productive cough, fever, night sweats, weight loss, chest pain, haemoptysis. CXR screening distinguishes between ‘any CXR abnormality’, which includes abnormalities that may not be considered suggestive of active or inactive TB and ‘abnormalities suggestive of TB’, which could be active or inactive TB. The two sequential screening algorithms that we consider are composed of ‘prolonged cough’ or ‘any TB symptom’ respectively as the first screen, and CXR as a second screening step for individuals with a positive first screen. Due to limited data, the accuracy of CXR is assumed to be the same in both algorithms, and reflects ‘any CXR abnormality’ in symptomatic persons [5, 13].

**Table 2:** **Model Parameters: sensitivity and specificity of screening methods and confirmatory tests**

Screen	Population (No. of studies)*	Sensitivity [95% CI]^†^	Specificity [95% CI]^†^	Reference
**Symptom screening**				
Prolonged Cough (2-3 weeks or longer)	Community TB prevalence surveys (8)	0.351 [0.244; 0.457]	0.947 [0.925; 0.968]	[5]
	SSA-high HIV prevalence^§^ (4)	0.492 [0.389; 0.597]	0.923 [0.891; 0.956]	[5]
	Asia-low HIV prevalence^§^ (4)	0.247 [0.176; 0.317]	0.963 [0.947; 0.979]	[5]
Any TB Symptom (out of 4-7 symptoms)	Combined (8)	0.770 [0.680; 0.860]	0.677 [0.502; 0.851]	[5]
	SSA-high HIV prevalence^§^ (4)	0.842 [0.756; 0.927]	0.740 [0.531; 0.949]	[5]
	Asia-low HIV prevalence^§^ (4)	0.698 [0.579; 0.818]	0.606 [0.347; 0.866]	[5]
**Chest X-ray screening**				
Any CXR abnormality	(3)	0.978 [0.951; 1.00]	0.754 [0.720; 0.788]	[5]
CXR abnormality suggestive of TB	(4)	0.868 [0.792; 0.945]	0.894 [0.867; 0.920]	[5]
**Chest X-ray screening as a 2nd screen**				
Any CXR abnormality	(1)	0.90 [0.81; 0.96]	0.56 [0.54; 0.58]	[5, 13]
**Confirmatory test**				
Sputum Smear microscopy	(30)	0.61 [0.31; 0.89]	0.98 [0.93; 1.0]	[14]
Xpert MTB/RIF	Multi-sites (1)	0.89 [0.63; 0.97]	0.99 [0.90; 1.00]	[15]
Clinical Diagnosis (PE), algorithm including trial of broad spectrum antibiotics and/or CXR and/or clinical judgment	Smear-negative presumptive TB patients from India, Uganda, South Africa, average of 3 sites, Lima	0.24 [0.10; 0.51]^‡^	0.94 [0.79; 0.97]^‡^	[16, 17]
Clinical Diagnosis (alternative) based on CXR highly consistent for TB	(1)	0.49 [0.45; 0.53]^‡^	0.90 [0.88; 0.92]^‡^	[18]

PE = point estimate; NPV = negative predictive value; SSA = Sub-Saharan Africa; TB = tuberculosis; CXR = chest X-ray.

*Number of studies included in the estimate.

^†^The values in between brackets reflect the 95% confidence interval, except for Xpert MTB/RIF the 95% prediction interval was used, and for SSM the range across studies (see Methods section).

^§^the 4 SSA-high HIV prevalence studies are from Zimbabwe, Zambia, South Africa and Kenya. The Asia-low HIV studies are from Vietnam, Myanmar, India and Cambodia.

^‡^An assumption is made that in an active screening program only a proportion of patients with a negative confirmatory SSM or Xpert MTB/RIF result receive clinical diagnosis, and this proportion depends on the NPV (rounded to 2 decimals as follows: (1-NPV)*10 If NPV ≥ 99.5% then the proportion is 5%. This is equivalent to multiplying the sensitivity parameter by (1-NPV)*10. The number of false-positive diagnoses is adjusted as follows: if S is the specificity parameter, the proportion of false-positives is [(1-S)*((1-NPV)*10)]. In algorithms 3 and 4 all persons with prolonged cough and a CXR abnormality and negative confirmatory tests are assumed to be further evaluated clinically.

### Confirmatory tests

As the first confirmatory tests we consider SSM which is widely available, or XP, whose availability is globally expanding. Since neither of these tests is 100% sensitive, clinical diagnosis, which implies a clinician’s judgment of the patient’s symptoms and signs and/or chest radiography findings, possibly re-assessed after a short course of broad-spectrum antibiotics, is a follow-on in some patients with a negative first test (in absence of mycobacterial culture). For XP we took the predicted sensitivity (89%) and specificity (99%) of a meta-analysis of XP as an initial test for TB detection replacing microscopy [15]. In sensitivity analyses we explored the predicted rather than the pooled 95% credible intervals for XP sensitivity and specificity, as a better reflection of what may happen in new situations. For the SSM sensitivity (Table 2) we use 61% as the point estimate (PE), which is both the unweighted average and median sensitivity of 30 studies included in two systematic reviews on sputum smear microscopy and processing techniques [14, 19]. In absence of a prediction interval obtained by meta-analysis we explored the range across included studies (31-89%) in sensitivity analysis, to accommodate the wide variation in patient characteristics, including HIV status, age, and disease severity, on the referral level where the test is done, on background epidemiology, on sputum processing and staining techniques, and skills and diagnostic quality [14, 19, 20]. The specificity PE (98%) is consistent with a number of reviews [14, 19, 20].

In clinical settings where mycobacterial cultures are not part of the routine diagnostic work-up, all or most patients who report with suggestive symptoms but have negative smears are expected to be evaluated clinically. In an active screening initiative we expect patients eligible for confirmatory testing to be less ill on average compared to patients who seek care themselves. We therefore assume that only a proportion of the persons with a positive screen but negative confirmatory test be further examined by clinical diagnosis [21], and that this proportion will depend on the probability of TB in a person with a negative SSM or XP result, i.e. the NPV at that point in the algorithm (Table 2), except for algorithms 3 and 4 where we assume that 100% of persons with prolonged cough and a CXR abnormality and negative confirmatory tests are further evaluated clinically. In the primary analysis the sensitivity and specificity of clinical diagnosis reflect settings where not all patients are examined by CXR [8, 16, 17]. In sensitivity analysis we explore the assumption about the proportion of patients receiving clinical evaluation (0-100%), and alternative parameter values for sensitivity and specificity based on a study where all clinically evaluated patients receive a diagnostic CXR and are considered a to have TB if the CXR is highly consistent for TB [18].

The sensitivity and specificity of all screens and confirmatory tests are relative to sputum culture as the reference standard, and considered to be independent. Simple decision trees were constructed in TreeAge software (TreeAge Software, Inc, Williamstown MA, USA) and model outputs were transferred to MS-Excel (Microsoft Corp, Seattle WA, USA) for further analysis. Ethical approval was not sought, as only secondary data were used.

For each algorithm we calculated the number of TP, FP, FN, and TN in categories A-F as described above, proportion of true TB cases detected, the PPV, NPV, NNS, the number of persons requiring confirmatory testing, and the number of screening CXRs, sputum microscopy examinations and XP tests required for different levels of TB prevalence in the population.

### Sensitivity analysis

In 10 scenarios (Table 3) we examine the effect of uncertainty in the model parameters on case detection, NNS, PPV, and resource requirements expressed as the number of CXRs, SSM and XP tests that needs to be done, assuming TB prevalence is 1%. We assume that if the sensitivity of a screening or confirmatory test increases to the extreme highest value of the uncertainty range, the specificity reduces to the lowest end of the uncertainty range. We also explore the effect of symptom screening using accuracy estimates from studies in African populations with high HIV prevalence versus Asian low HIV settings. Since real life experience with XP is less compared to SSM, we examine the effect if the specificity of smear microscopy and XP were the same (98%) in practice [14, 20, 22].

**Table 3:** **Scenarios to examine the effect of uncertainty in the model parameters**

Variation in	Scenario	Reference
1. Accuracy of screening tests (symptom, CXR)	a. High sensitivity, low specificity*	[5]
	b. Low sensitivity, high specificity*	[5]
2. Accuracy of confirmatory tests (SSM, XP)	a. High sensitivity, low specificity*	[14, 15, 19, 20]
	b. Low sensitivity, high specificity*	[14, 15, 19, 20]
3. Specificity of SSM, XP	Assume 98% specificity for SSM and XP	[22]
4. Proportion of persons with negative SSM or XP receiving clinical diagnosis	a. 0%	
	b. 100%	
5. Accuracy of clinical diagnosis	Entirely based on CXR diagnosis	[18]
6. Accuracy of screening tests in different settings	a. Sub Saharan Africa/high HIV-population	[5]
	b. Asia/low HIV population	[5]

*set to the extremes of the ranges shown in Table 1.

## Results

### Sensitivity and NNS

**Figure 3:**
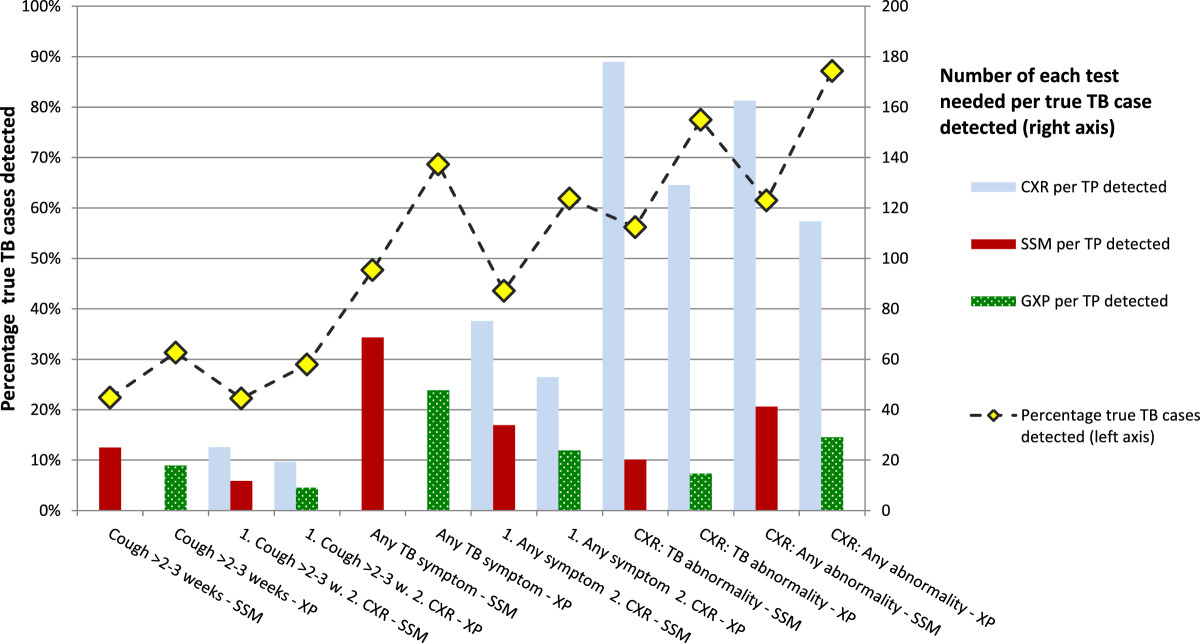
**TB cases detection and requirements for screening chest X-rays and confirmatory tests of each algorithm, assuming 1% TB prevalence among the screened population.** CXR = chest X-ray for screening; SSM = sputum smear microscopy; XP = Xpert MTB/RIF; TP = true positive; 1 = first screen; 2 = second screen if the first screen is positive.

**Figure 4:**
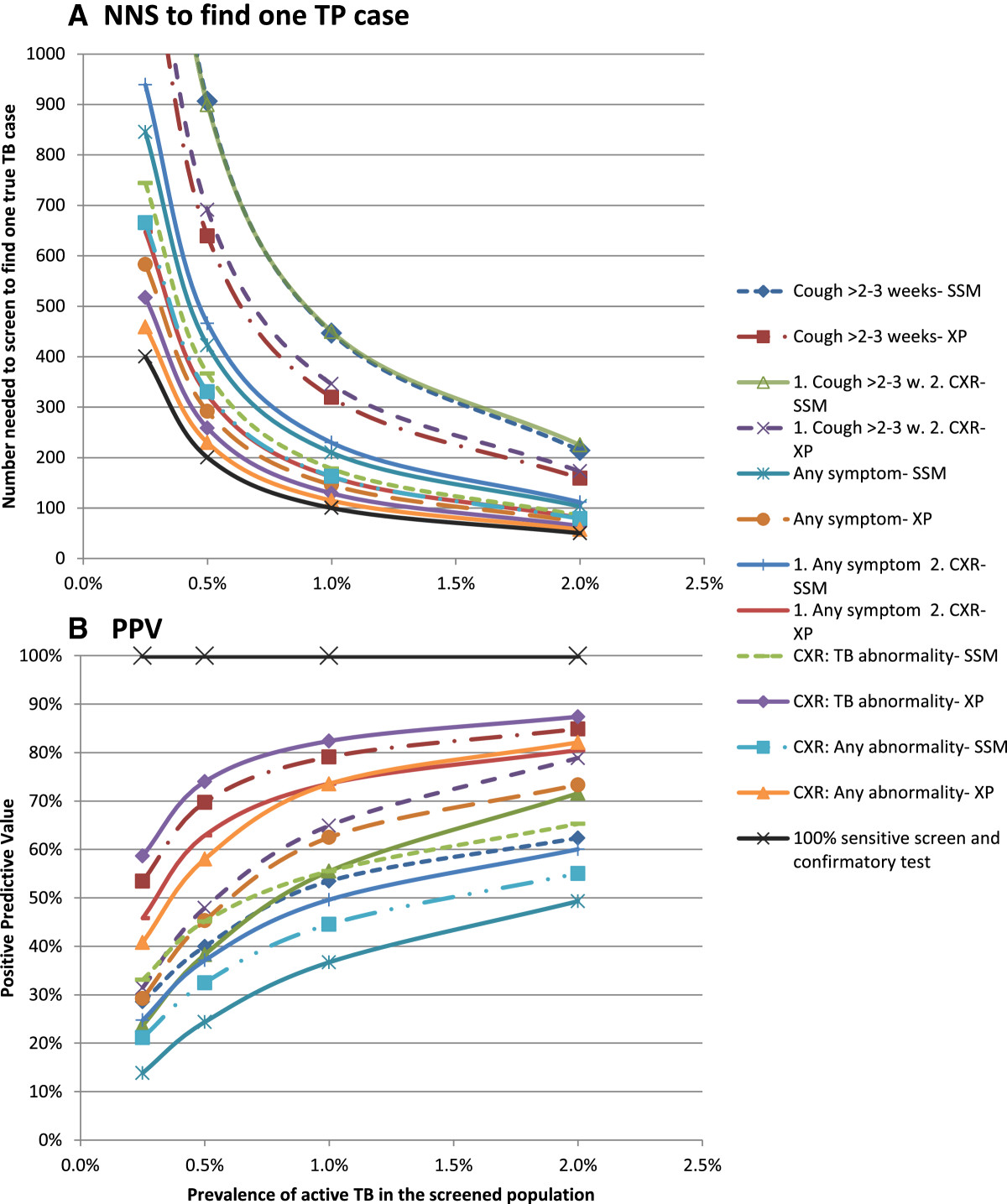
**Number needed to screen to find one true case of active TB and positive predictive value of each algorithm at different levels of TB prevalence.** Panel **A**: Number needed to screen (NNS) to find one true positive (TP) case; Panel **B**: Positive predictive value (PPV). CXR = chest X-ray for screening; SSM = sputum smear microscopy; XP = Xpert MTB/RIF; 1 = first screen; 2 = second screen if first is positive.

If prolonged cough, which has low sensitivity, is either the only screen or a first step followed by CXR, the proportion of true TB cases detected is low: 22% if the confirmatory test for screen-positives is SSM, and approximately 30% if XP (Figure 3). The NNS to find a true TB case depends on the sensitivity of the algorithm and increases with decreasing TB prevalence (Figure 4A). Of algorithms using screening for prolonged cough the NNS is 3-4 times higher compared to the NNS of the most sensitive algorithm and exceeds 1000 if the TB prevalence is below 0.5%. The highest case detection and lowest NNS is achieved if XP is used for confirmatory testing, and screening is either by CXR for any abnormality (87%) or TB abnormalities (77%), or by screening for any TB symptom (69%).

Detailed information on the outcomes of the screening and confirmatory testing is available in the Additional file 1: Table S1. For each of the 12 algorithm the number of TP, FP, TN, FN, PPV, NPV and the number of tests required for an assumed prevalence in the population to be screened of 0.5, 1.0 and 2.0% are provided with and without clinical diagnosis following a negative SSM or XP.

### Specificity and PPV

The algorithms in which the specificity of the screening test and/or the confirmatory test(s) is lowest result in more false positive diagnoses and thus in a lower PPV, especially if the TB prevalence is low and/or the sensitivity of the algorithm is low (Figure 4B). If the TB prevalence is 0.5% only four algorithms attain a PPV greater than 50% (which means that the number of TP cases exceeds the number of FPs). Those are algorithms that use XP as the confirmatory test, and screen with CXR for either any abnormality or TB abnormalities, symptom screening for prolonged cough, or sequential screening with any symptom followed by CXR. The PPV of screening for any symptom followed by SSM does not reach 50% even if the TB prevalence is 2%.

### Requirements in terms of diagnostic tests

Of the algorithms with high sensitivity, CXR screening for TB abnormalities followed by XP requires 15 XP tests per TB case detected when TB prevalence is 1% (Figure 3). This is more efficient compared to any CXR abnormality (29 XP tests), due to better specificity. When counting the number of screening CXRs needed per one TP case, CXR screening for TB abnormalities requires 129 CXRs and is slightly less efficient compared to CXR screening for any abnormality (115 CXRs), due to lower sensitivity. Screening for any TB symptom results in high requirements for confirmatory tests per TP case (69 SSM or 48 XP). The efficiency of symptom screening improves if CXR as a second screen is added for symptom screen positives, but adds the need for CXRs compared to symptom screening alone. The number of tests per TP approximately doubles if prevalence halves.

### Sensitivity analyses

**Figure 5:**
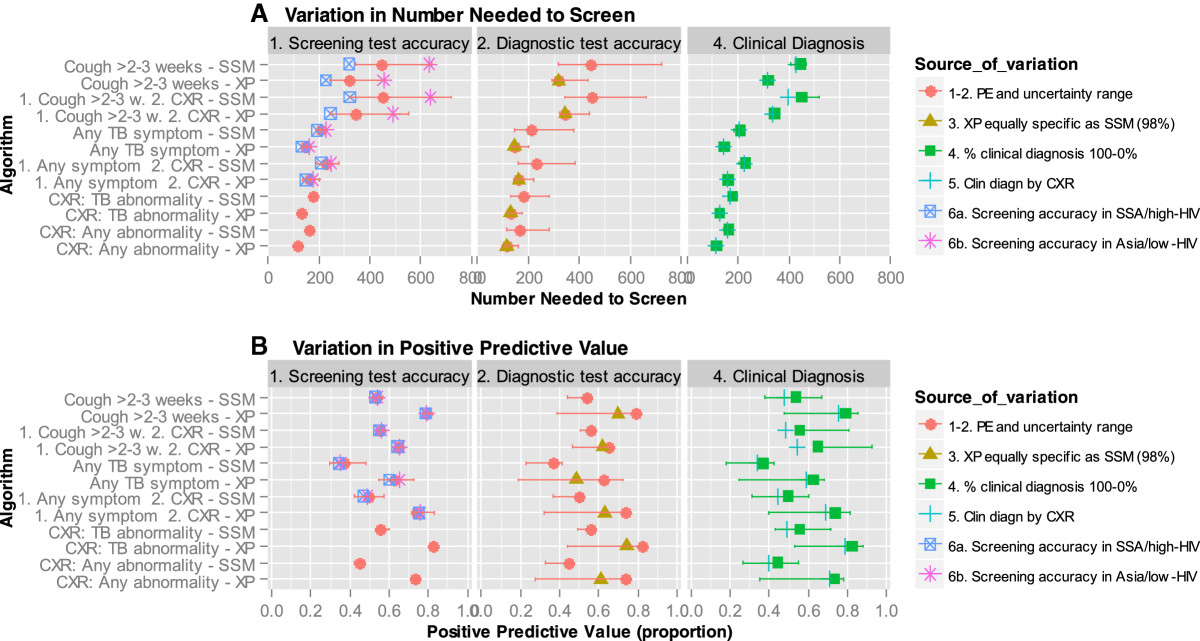
**Effect of uncertainty in the accuracy of screening and diagnostic tests and assumptions about clinical diagnosis on the NNS and PPV, assuming 1% TB prevalence in the screened population.** Panel **A**: Variation in the number needed to screen (NNS); Panel **B**: Variation in the positive predictive value (PPV). The symbols represent the point estimates and the vertical bars the range due to uncertainty in the model parameter, as specified in Table 2. The specific scenarios are listed in Table 3. CXR = chest X-ray for screening; SSM = sputum smear microscopy; XP = Xpert MTB/RIF; 1 = first screen; 2 = second screen if first is positive. SSA = sub Saharan Africa.

Uncertainty and setting-specific variation in the sensitivity of symptom screening, translates into considerable variation in the NNS of algorithms with low sensitivity, i.e. those screening with prolonged cough (Figure 5). If the sensitivity of the cough screen is at the low end, the NNS is double compared to sensitivity at the high end of the range. For screening with any TB symptom the PPV is more susceptible to uncertainty. Setting-specific variation in the sensitivity and specificity of symptom screening does not change the ranking of the NNS or PPV of the 12 algorithms. The assumptions about clinical diagnosis have little effect on the NNS. The PPV of all algorithms drops if the proportion of persons receiving clinical diagnosis after a negative confirmatory test would increase or a clinical diagnostic algorithm with higher sensitivity but lower specificity would be applied (due to an increase in false positive diagnoses). The largest increase in the number of confirmatory tests required per TP case detected is caused by a lower sensitivity of the confirmatory test (Additional file 2: Table S2).

## Discussion

This modeling study shows how algorithms that could be considered for a TB screening initiative differ in the proportion of TB cases detected, the NNS, the risk of providing a TB diagnosis to individuals who do not have TB, an indication of the number of tests that need to be used to detect a true case of TB, and how those are affected by the prevalence of TB in the screened population and by variation in performance of screening and diagnostic tests.

### How could TB screening programs use this information

For a TB screening program in a specific setting, the choice of screening and diagnostic methods will depend on several considerations. The choice between symptom and CXR screening may be driven by the availability, cost and mobility of CXR equipment, but should be balanced against the overall resource requirements for an active screening initiative and the expected yield. The algorithm composed of screening for prolonged cough followed by smear microscopy requires the least resources for screening and confirmatory tests, but the low case detection, only 22% of the TB cases, results in a high NNS which is inefficient. In severely resource constrained settings this algorithm may nevertheless be chosen since a low number of confirmatory tests is needed, it does not require expensive equipment, and the costs of reaching out to the screened population may be low in such settings. A more sensitive symptom screen, any TB symptom, increases the proportion of cases detected and thus lowers the NNS, but creates a large number of persons requiring confirmatory testing and a low PPV due to an expected increase in FP diagnoses. The latter creates a treatment burden for patients and the health service. An additional disadvantage of symptom screening and especially prolonged cough, is the wide variation in accuracy observed between studies [5], which was in part explained by differences in region and population HIV-prevalence. As a result the NNS in a specific setting may be approximately 50% higher or lower than the average that we used. Less variation is expected from CXR screening.

In settings where the prevalence of TB is low, the NNS is high for all algorithms, even for an algorithm with 100% sensitivity, resulting in high costs of screening per TP TB case found, and the PPV is low. Therefore in low prevalence settings great caution is needed and high specificity is very important. Mechanisms to improve specificity should be considered, for instance adding confirmatory culture before TB treatment is initiated in patients with absent or minimal symptoms, or XP may be used to confirm positive SSM. Target groups should be selected for whom it is critically important to detect active TB, and/or exclude active TB because of possible eligibility for treatment of latent TB infection (LTBI), e.g. HIV-infected [23]. Under those conditions some overtreatment of active TB may be more acceptable. In contrast, when the TB prevalence in the population to be screened is 2% or above, the NNS is much lower and the PPV is higher for all algorithms. Very high specificity is less crucial compared to low prevalence settings. These clinical epidemiological principles do not only guide the choice for an algorithm, but also provide a key rationale to focus screening to highest risk groups where TB prevalence may even be above 2% like HIV-infected, contacts of infectious TB cases, persons with silicosis, prisoners [24], and the elderly population in some very-high TB burden countries in Asia [25, 26].

CXR screening becomes much more attractive when XP is available. CXR for screening should ideally not be combined with SSM for diagnosis because of an increase in the NNS by approximately half, and the higher risk of false positives, especially if among SSM negatives the CXR is also used for a clinical diagnosis. Although CXR is good for screening because of high sensitivity, it is not as good for diagnosis since a CXR based clinical diagnosis has low specificity [18]. CXR screening has additional benefits. Persons with highly suggestive TB abnormalities can be identified who are missed by bacteriological tests. These persons are at increased risk of developing active TB in the future and would benefit from follow-up and possibly preventive treatment [27]. Second, the ability to detect other diseases. These advantages only apply if the health service has the capacity to provide follow-up of patients with unclear TB outcomes or clinical services for other identified conditions. A screening program should consider all such possible outcomes and consequences, and include those in informed consent to TB screening [4].

The cost and cost-effectiveness of an algorithm will be an important consideration, which we do not address in this study. CXR and XP are more costly tests compared to symptom screening and SSM [28]. However, digital CXR has very low running cost and is an attractive tool if the technology is already available, or if the high start-up costs could be covered under investments for general improvements of diagnostic capacity of a health system. Cost for screening program operations will be very setting specific. Ultimately cost-effectiveness also depends on the effect that different algorithms may have on transmission and at which interval they should be applied to accelerate or sustain reduced transmission. The development of a combined transmission and cost-effectiveness model will be important to compare screening and diagnostic strategies. However, empirical data on the effect of screening or active case finding on transmission are scarce [29]. Field studies comparing the effectiveness and cost-effectiveness of different algorithms and screening intervals are needed to better inform decision makers and screening models.

We used a simple model to focus on the most important concepts and show the expected variation from uncertainty and variation in test accuracy, which has limitations. The sensitivity of the confirmatory test are based on clinical studies [30, 31], and is generally lower in early case detection, like in prevalence surveys [6, 26, 32]. We assumed that the accuracy of screening and confirmatory tests are independent, while those may correlate in practice. Also the yield of screening may diminish if repeated at regular intervals [33]. As the sensitivity analysis points out, considerable variation in the NNS can be expected from variation in the sensitivity of the screening and confirmatory tests. Variability depends on the population tested [14, 32], but for SSM, also on technique and skills in getting a good sample, preparation, and reading. Quality assurance is therefore important. The extent and accuracy of clinical diagnosis, if applied in absence of mycobacterial culture may also vary greatly. Possible effects on participation of perceptions of the population to be screened are not considered in this model. A screening program that only identifies a small proportion of the TB cases may not be as reassuring for persons with a negative test result. Screening tests with low specificity may either cause unnecessary worry, or decrease motivation since one rarely receives a positive confirmatory test.

Our analysis points to a number of monitoring and evaluation questions. An assessment of the true proportion of TB cases detected would be desirable, but will often not be feasible. Instead an estimation of the specificity of the screen, the NNS and PPV could be obtained from routine monitoring data on participation in the screening program, the numbers and proportions of persons testing positive on screening and of TB cases diagnosed, and by periodic retesting of the persons diagnosed with TB with a more sensitive method (culture). If these indicators are outside the expected range for the algorithm used and presumed TB prevalence, more in-depth evaluation may be needed. Future research should address the need for empirical data and cost-effectiveness studies, and also focus on the development of a better screening test. Digital CXR has very low running costs and automated reading may be an option in the future [34, 35]. However, a simple, more portable screening test with greater and more consistent accuracy compared to symptom screening could greatly enhance TB screening.

This analysis concerns screening scenarios, but has relevance for diagnostic algorithms among people actively seeking care with TB symptoms as well. CXR may be used to triage patients for further bacteriological tests [36]. XP as a replacement of SSM will not only increase sensitivity and thus increase case detection, but also increase the PPV.

## Conclusion

In conclusion, this study provides guidance on choice of algorithms, keeping in mind that with currently available means ‘the one ideal algorithm’ does not exist. Generally an algorithm composed of CXR screening followed by confirmatory testing with XP can achieve the lowest NNS and highest PPV, and the validity is least amendable to setting-specific variation. However resource requirements for tests and equipment may be prohibitive in some settings and a reason to opt for symptom screening and SSM. To better inform disease control programs about TB screening options we need empirical data to confirm the modeled yield, cost-effectiveness studies, transmission models and a better screening test.

## Electronic supplementary material

Additional file 1: Table S1: Detailed information on the outcomes of the screening and confirmatory testing. (XLSX 48 KB)

Additional file 2: Table S2: Sensitivity Analysis, showing the effect of variation in model parameters on five indicators, for a prevalence of 1.0%. (DOC 126 KB)

Below are the links to the authors’ original submitted files for images.

Authors’ original file for figure 1

Authors’ original file for figure 2

Authors’ original file for figure 3

Authors’ original file for figure 4

Authors’ original file for figure 5
