# Supplementary material for: Choosing algorithms for TB screening: a modelling study to compare yield, predictive value and diagnostic burden
Source: BMC Infect Dis. 2014 Oct 19;14:532. doi: 10.1186/1471-2334-14-532 (PMC4287425; doi:10.1186/1471-2334-14-532)
Supplement: Supplementary file 2 — Additional file 2: Table S2: Sensitivity Analysis, showing the effect of variation in model parameters on five indicators, for a prevalence of 1.0%. (DOC 126 KB) [file 12879_2014_4008_MOESM2_ESM.doc]

**Additional file 2: Table S2 (online only): Sensitivity Analysis, showing the effect of variation in model parameters on five indicators, for a prevalence of 1.0%**.

| Screening method | Conf. Test | Indicator | Point Estimate | Scenario number (as listed in Text box 2) | | | | | | | | | |
| --- | --- | --- | --- | --- | --- | --- | --- | --- | --- | --- | --- | --- | --- |
|  |  |  |  | 1.a | 1.b | 2.a | 2.b | 3. | 4.a | 4.b | 5 | 6.a | 6.b |
| Cough >2-3 weeks | SSM | Percent of true cases detected | 22% | 29% | 16% | 31% | 14% | 22% | 21% | 25% | 23% | 31% | 16% |
|  |  | NNS to find one TP case | 446 | 343 | 642 | 319 | 725 | 446 | 467 | 405 | 427 | 318 | 634 |
|  |  | Positive predictive value | 53% | 51% | 57% | 44% | 47% | 53% | 67% | 38% | 47% | 53% | 54% |
|  |  | No. of screening CXR per TP | 0 | 0 | 0 | 0 | 0 | 0 | 0 | 0 | 0 | 0 | 0 |
|  |  | No. of SSM per TP | 25 | 27 | 22 | 18 | 41 | 25 | 26 | 23 | 24 | 26 | 25 |
| Cough >2-3 weeks | XP | Percent of true cases detected | 31% | 41% | 22% | 34% | 23% | 31% | 31% | 32% | 31% | 44% | 22% |
|  |  | NNS to find one TP case | 319 | 245 | 459 | 294 | 434 | 319 | 320 | 311 | 318 | 228 | 454 |
|  |  | Positive predictive value | 79% | 78% | 81% | 39% | 71% | 70% | 86% | 47% | 75% | 78% | 79% |
|  |  | No. of screening CXR per TP | 0 | 0 | 0 | 0 | 0 | 0 | 0 | 0 | 0 | 0 | 0 |
|  |  | No. of XP per TP | 18 | 19 | 16 | 16 | 24 | 18 | 18 | 17 | 18 | 18 | 18 |
| 1. Cough >2-3 w. 2. CXR | SSM | Percent of true cases detected | 22% | 31% | 14% | 29% | 15% | 22% | 19% | 22% | 25% | 31% | 16% |
|  |  | NNS to find one TP case | 450 | 324 | 719 | 345 | 665 | 450 | 519 | 450 | 395 | 321 | 639 |
|  |  | Positive predictive value | 55% | 54% | 58% | 50% | 53% | 55% | 81% | 55% | 48% | 55% | 56% |
|  |  | No. of screening CXR per TP | 25 | 26 | 25 | 19 | 37 | 25 | 29 | 25 | 22 | 26 | 25 |
|  |  | No. of SSM per TP | 12 | 12 | 11 | 9 | 17 | 12 | 14 | 12 | 10 | 12 | 12 |
| 1. Cough >2-3 w. 2. CXR | XP | Percent of true cases detected | 29% | 40% | 18% | 31% | 23% | 29% | 28% | 29% | 30% | 41% | 20% |
|  |  | NNS to find one TP case | 345 | 249 | 552 | 324 | 440 | 345 | 356 | 345 | 335 | 246 | 491 |
|  |  | Positive predictive value | 65% | 63% | 67% | 47% | 63% | 62% | 92% | 65% | 54% | 64% | 65% |
|  |  | No of screening CXR per TP | 19 | 20 | 19 | 18 | 25 | 19 | 20 | 19 | 19 | 20 | 19 |
|  |  | No of XP per TP | 9 | 10 | 8 | 9 | 12 | 9 | 9 | 9 | 9 | 9 | 9 |
| Any symptom | SSM | Percent of true cases detected | 48% | 53% | 43% | 69% | 26% | 48% | 47% | 54% | 48% | 52% | 44% |
|  |  | NNS to find one TP case | 210 | 188 | 234 | 146 | 378 | 210 | 213 | 185 | 206 | 192 | 228 |
|  |  | Positive predictive value | 37% | 30% | 48% | 23% | 41% | 37% | 42% | 18% | 34% | 34% | 35% |
|  |  | No. of screening CXR per TP | 0 | 0 | 0 | 0 | 0 | 0 | 0 | 0 | 0 | 0 | 0 |
|  |  | No. of SSM per TP | 69 | 94 | 36 | 48 | 124 | 69 | 70 | 60 | 68 | 76 | 60 |
| Any symptom | XP | Percent of true cases detected | 69% | 77% | 61% | 75% | 49% | 69% | 69% | 71% | 69% | 75% | 62% |
|  |  | NNS to find one TP case | 146 | 130 | 165 | 134 | 203 | 146 | 146 | 142 | 145 | 133 | 161 |
|  |  | Positive predictive value | 62% | 55% | 72% | 19% | 72% | 48% | 68% | 25% | 59% | 60% | 65% |
|  |  | No. of screening CXR per TP | 0 | 0 | 0 | 0 | 0 | 0 | 0 | 0 | 0 | 0 | 0 |
|  |  | No. of XP per TP | 48 | 65 | 25 | 44 | 67 | 48 | 48 | 46 | 48 | 53 | 42 |
| 1. Any symptom 2. CXR | SSM | Percent of true cases detected | 44% | 52% | 36% | 62% | 26% | 44% | 42% | 49% | 45% | 48% | 40% |
|  |  | NNS to find one TP case | 229 | 193 | 280 | 162 | 383 | 229 | 237 | 205 | 223 | 210 | 249 |
|  |  | Positive predictive value | 50% | 42% | 57% | 37% | 44% | 50% | 60% | 31% | 45% | 47% | 49% |
|  |  | No. of screening CXR per TP | 75 | 97 | 43 | 53 | 126 | 75 | 77 | 67 | 73 | 84 | 66 |
|  |  | No. of SSM per TP | 34 | 45 | 19 | 24 | 57 | 34 | 35 | 30 | 33 | 38 | 30 |
| 1. Any symptom 2. CXR | XP | Percent of true cases detected | 62% | 74% | 49% | 67% | 45% | 62% | 62% | 64% | 62% | 68% | 56% |
|  |  | NNS to find one TP case | 162 | 136 | 203 | 149 | 223 | 162 | 162 | 157 | 161 | 148 | 178 |
|  |  | Positive predictive value | 74% | 72% | 83% | 32% | 73% | 63% | 81% | 40% | 69% | 75% | 76% |
|  |  | No. of CXR per TP | 53 | 68 | 31 | 49 | 73 | 53 | 53 | 52 | 53 | 59 | 47 |
|  |  | No. of XP per TP | 24 | 32 | 14 | 22 | 33 | 24 | 24 | 23 | 24 | 27 | 21 |
| CXR: TB abnormality | SSM | Percent of true cases detected | 56% | 60% | 51% | 77% | 36% | 56% | 53% | 61% | 60% | 56% | 56% |
|  |  | NNS to find one TP case | 178 | 166 | 195 | 129 | 281 | 178 | 189 | 164 | 168 | 178 | 178 |
|  |  | Positive predictive value | 56% | 55% | 60% | 49% | 49% | 56% | 72% | 43% | 49% | 56% | 56% |
|  |  | No. of screening CXR per TP | 178 | 166 | 195 | 129 | 281 | 178 | 189 | 164 | 168 | 178 | 178 |
|  |  | No. of SSM per TP | 20 | 23 | 17 | 15 | 32 | 20 | 21 | 19 | 19 | 20 | 20 |
| CXR: TB abnormality | XP | Percent of true cases detected | 77% | 84% | 71% | 84% | 57% | 77% | 77% | 80% | 78% | 77% | 77% |
|  |  | NNS to find one TP case | 129 | 119 | 141 | 119 | 175 | 129 | 129 | 126 | 129 | 129 | 129 |
|  |  | Positive predictive value | 82% | 80% | 81% | 44% | 76% | 74% | 88% | 53% | 79% | 82% | 82% |
|  |  | No. of screening CXR per TP | 129 | 119 | 141 | 119 | 175 | 129 | 129 | 126 | 129 | 129 | 129 |
|  |  | No. of XP per TP | 15 | 17 | 12 | 13 | 20 | 15 | 15 | 14 | 15 | 15 | 15 |
| CXR: Any abnormality | SSM | Percent of true cases detected | 61% | 63% | 60% | 87% | 35% | 61% | 60% | 69% | 63% | 61% | 61% |
|  |  | NNS to find one TP case | 163 | 159 | 167 | 115 | 284 | 163 | 168 | 145 | 158 | 163 | 163 |
|  |  | Positive predictive value | 45% | 42% | 48% | 33% | 45% | 45% | 55% | 27% | 40% | 45% | 45% |
|  |  | No. of screening CXR per TP | 163 | 159 | 167 | 115 | 284 | 163 | 168 | 145 | 158 | 163 | 163 |
|  |  | No. of SSM per TP | 41 | 46 | 37 | 29 | 72 | 41 | 42 | 37 | 40 | 41 | 41 |
| CXR: Any abnormality | XP | Percent of true cases detected | 87% | 89% | 85% | 95% | 63% | 87% | 87% | 90% | 87% | 87% | 87% |
|  |  | NNS to find one TP case | 115 | 112 | 118 | 105 | 158 | 115 | 115 | 112 | 115 | 115 | 115 |
|  |  | Positive predictive value | 74% | 71% | 72% | 28% | 69% | 61% | 78% | 35% | 71% | 74% | 74% |
|  |  | No. of screening CXR per TP | 115 | 112 | 118 | 105 | 158 | 115 | 115 | 112 | 115 | 115 | 115 |
|  |  | No. of XP per TP | 29 | 32 | 26 | 27 | 40 | 29 | 29 | 28 | 29 | 29 | 29 |
| Conf. Test=confirmatory test ; No.=number; CXR=chest X-ray for screening ; TP=true positive ; NNS=number needed to screen ; SSM=sputum smear microscopy ; XP=Xpert MTB/RIF | | | | | | | | | | | | | |
