# Supplementary material for: Choosing algorithms for TB screening: a modelling study to compare yield, predictive value and diagnostic burden
Source: BMC Infect Dis. 2014 Oct 19;14:532. doi: 10.1186/1471-2334-14-532 (PMC4287425; doi:10.1186/1471-2334-14-532)
Supplement: Supplementary file 3 — Authors’ original file for figure 1 [file 12879_2014_4008_MOESM3_ESM.pdf]

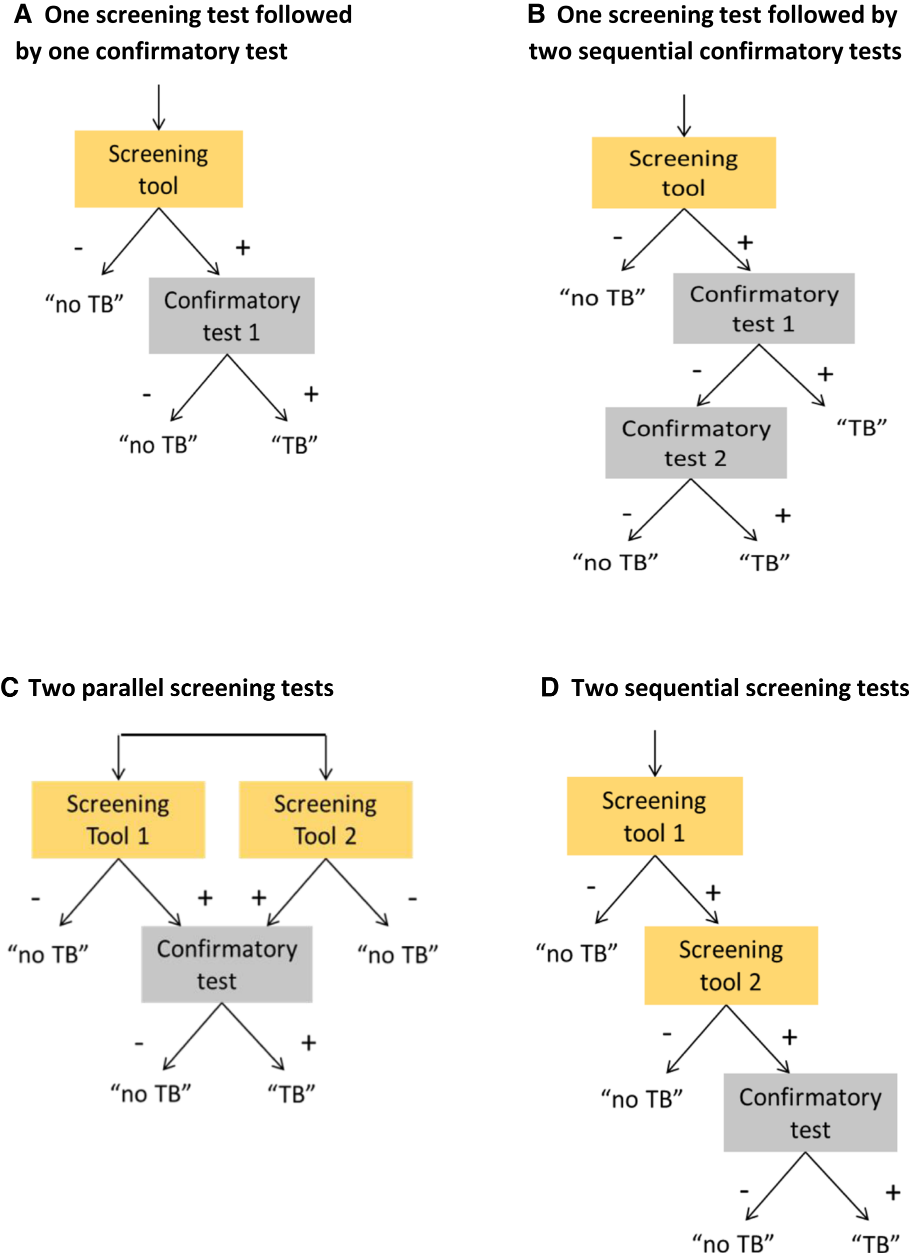
A One screening test followed by one confirmatory test
Screening tool
-
+
"no TB"
Confirmatory test 1
-
+
"no TB"
"TB"
B One screening test followed by two sequential confirmatory tests
Screening tool
-
+
"no TB"
Confirmatory test 1
-
+
Confirmatory test 2
"TB"
-
+
"no TB"
"TB"
C Two parallel screening tests
Screening Tool 1
Screening Tool 2
-
+
+
-
"no TB"
Confirmatory test
"no TB"
-
+
"no TB"
"TB"
D Two sequential screening tests
Screening tool 1
-
+
"no TB"
Screening tool 2
-
+
"no TB"
Confirmatory test
-
+
"no TB"
"TB"
