# Supplementary figures and images for: Choosing algorithms for TB screening: a modelling study to compare yield, predictive value and diagnostic burden
Source: BMC Infect Dis. 2014 Oct 19;14:532. doi: 10.1186/1471-2334-14-532 (PMC4287425; doi:10.1186/1471-2334-14-532)

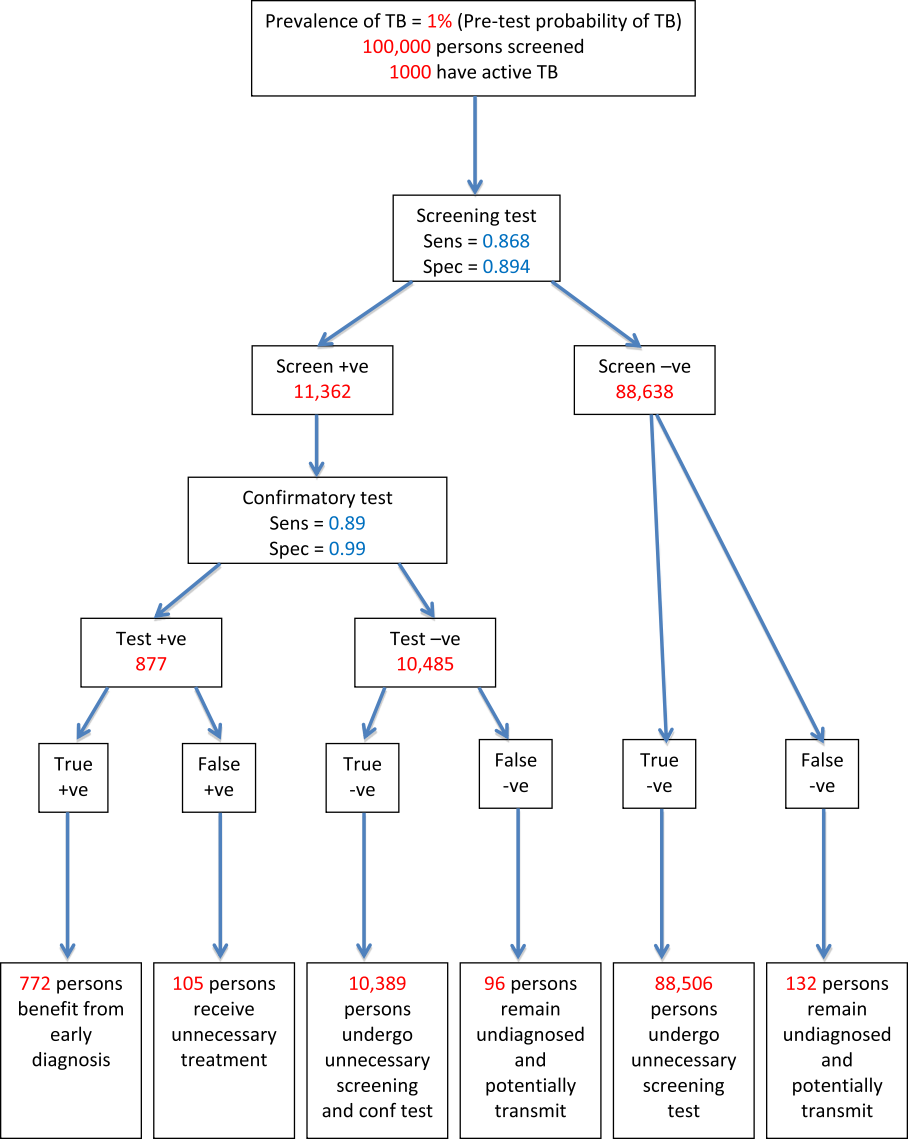

Supplement: Supplementary file 4 — Authors’ original file for figure 2 [file 12879_2014_4008_MOESM4_ESM.pdf]

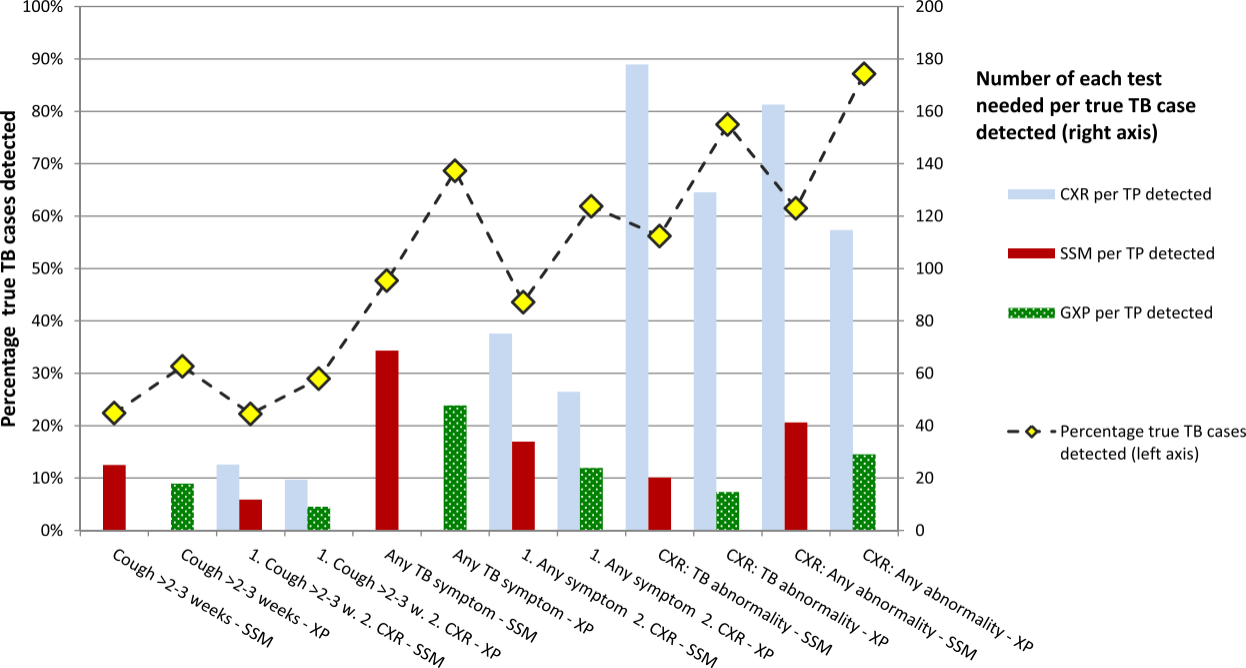

Supplement: Supplementary file 5 — Authors’ original file for figure 3 [file 12879_2014_4008_MOESM5_ESM.pdf]

# A NNS to find one TP case

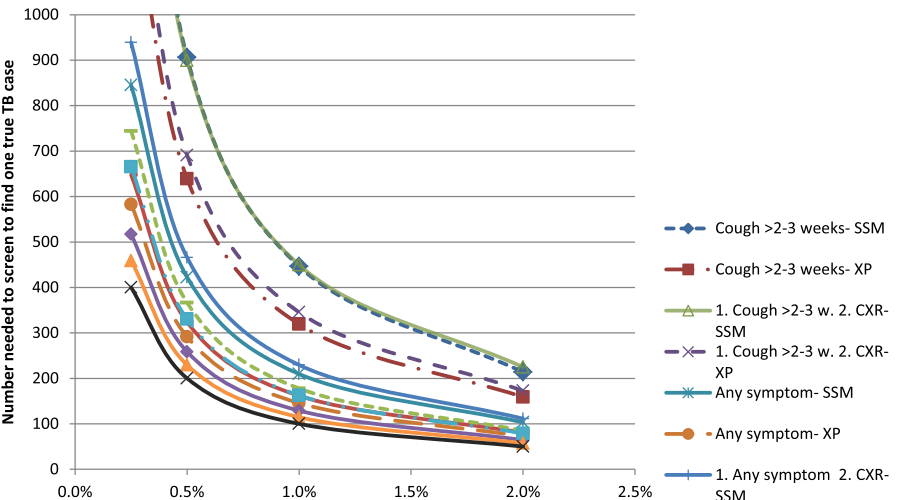

# B PPV

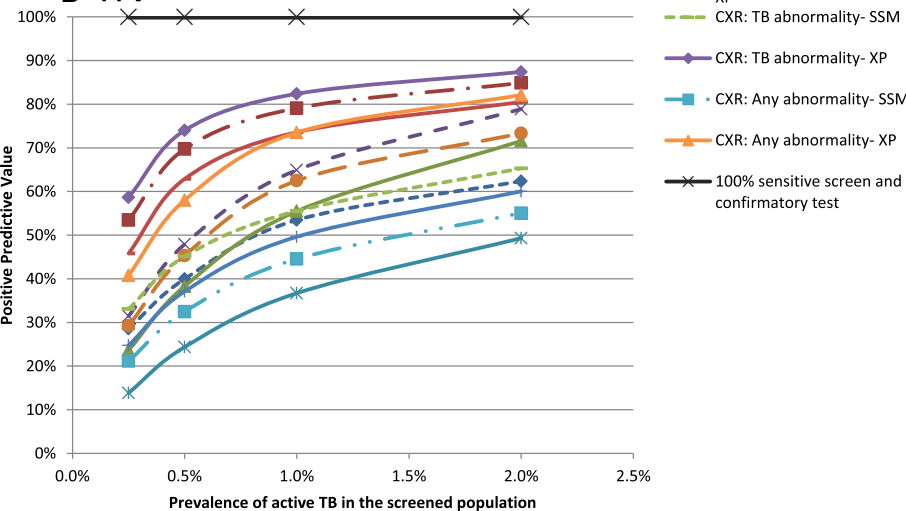

Supplement: Supplementary file 6 — Authors’ original file for figure 4 [file 12879_2014_4008_MOESM6_ESM.pdf]

## A Variation in Number Needed to Screen

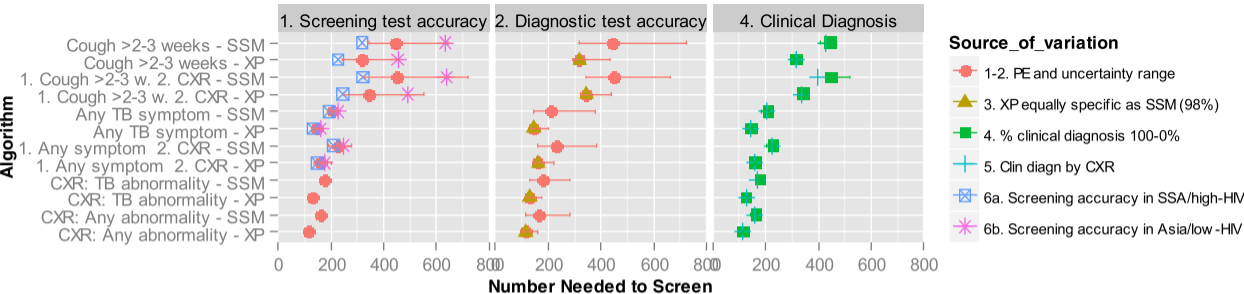

## B Variation in Positive Predictive Value

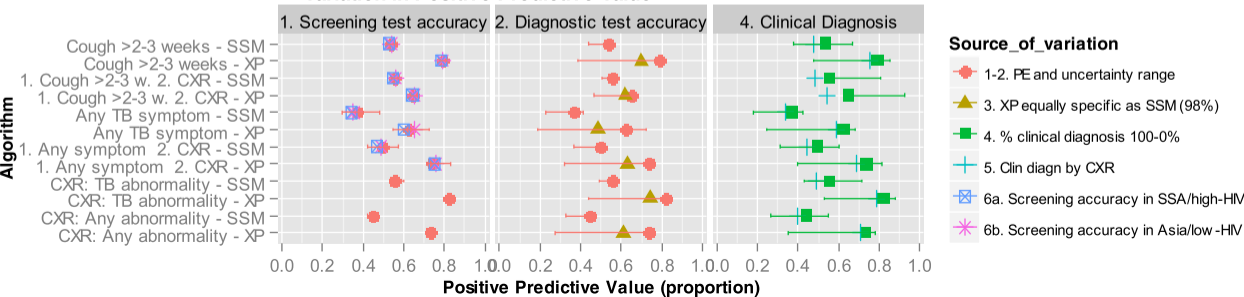

Supplement: Supplementary file 7 — Authors’ original file for figure 5 [file 12879_2014_4008_MOESM7_ESM.pdf]
